# Corneal endothelial changes in type 2 diabetes mellitus: a systematic review and meta-analysis with subgroup comparisons by diabetic retinopathy status and disease duration

**DOI:** 10.3389/fendo.2026.1982467

**Published:** 2026-09-29

**Authors:** Yongran Li, Chenlin Li, Zhimin Cen, Dingwang Su, Tulong Lin

**Affiliations:** 1 Center of Ophthalmology, Heyou Hospital, Foshan, China; 2 Department of Cardiology, Jieyang People’s Hospital, Jieyang, China; 3 Center of Ophthalmology, Zhongshan Torch Development Zone People’s Hospital, Zhongshan, China

**Keywords:** corneal endothelium, diabetic retinopathy, meta-analysis, specular microscopy, type 2 diabetes mellitus

## Abstract

**Background:**

Type 2 diabetes mellitus (T2DM) affects multiple ocular structures, yet the extent of corneal endothelial alterations across different stages of diabetic retinopathy (DR) and disease duration remains unclear. This meta-analysis quantified corneal endothelial changes in T2DM across subgroups defined by DR status and diabetes duration.

**Methods:**

This systematic review adhered to PRISMA and MOOSE guidelines (PROSPERO: CRD420261473313). Five databases were searched from inception through July 15, 2026. Cross-sectional studies comparing endothelial cell density (ECD), coefficient of variation (CV), and percentage of hexagonal cells (HEX) in T2DM patients versus controls or between T2DM subgroups were included. Study quality was assessed using the AHRQ checklist. Effect sizes were calculated as Cohen’s d using fixed-effect or random-effects models based on heterogeneity. Sensitivity analyses used the leave-one-out method; publication bias was assessed with funnel plots, Egger’s test, and trim-and-fill analysis.

**Results:**

Twenty-two studies (5,878 eyes) were included. T2DM patients showed significantly lower ECD (d = −0.35, P < 0.001), higher CV (d = 0.25, P < 0.001), and lower HEX (d = −0.28, P < 0.001) versus controls. Eyes with DR had lower ECD (d = −0.22, P = 0.002), higher CV (d = 0.26, P = 0.005), and lower HEX (d = −0.23, P = 0.02) versus eyes without DR. No significant differences emerged between PDR and NPDR. Longer disease duration (≥10 years) was associated with lower ECD (d = −0.39, P = 0.04) and lower HEX (d = −0.18, P = 0.003). Sensitivity analyses showed that HEX (T2DM vs. controls), CV (PDR vs. NPDR), and CV (duration comparison) were not robust; trim-and-fill reversed the CV duration result.

**Conclusions:**

T2DM is associated with impaired corneal endothelial morphology, with greater changes in the presence of DR and longer duration. Several estimates were sensitive to individual studies or publication bias, warranting cautious interpretation. Preoperative endothelial assessment may be beneficial in diabetic patients, particularly those with DR or long disease duration.

**Systematic review registration:**

https://www.crd.york.ac.uk/PROSPERO/view/CRD420261473313, identifier CRD420261473313.

## 1 Background

Diabetes mellitus (DM) is a significant global public health problem, with more than 500 million individuals living with this metabolic disorder worldwide (1). Type 2 DM (T2DM) is the predominant clinical subtype, accounting for 96% of all diabetic cases, and its prevalence continues to rise rapidly alongside aging populations, sedentary lifestyles and rising obesity rates across Asian, Middle Eastern and Western nations (2). The eye ranks among the most vulnerable target organs of diabetic damage, attributed to dense microvascular networks and high basal metabolic demands of intraocular tissues. Owing to the vast T2DM patient population, diabetes associated ocular complications place substantial strain on global healthcare systems. Diabetic retinopathy (DR) is the most widely recognized ocular complication of diabetes mellitus and serves as a good indicator of the state of microvascular disease in the rest of the organs. Beyond retinal lesions, T2DM also has profound clinically relevant effects on the ocular surface. Hyperglycemia and formation of advanced glycation end products damage multiple corneal layers, including the epithelium, corneal nerves, immune cells and endothelium (3, 4). A broad spectrum of pathological manifestations can arise from diabetes-related corneal injury, such as reduced corneal sensitivity, delayed re-epithelialization following corneal abrasion, basement-membrane abnormalities, corneal neuropathy and endothelial decompensation (5, 6). Corneal endothelial injury in diabetes is often insidious, presenting without obvious subjective symptoms in the early stage and therefore easily overlooked by clinicians. Under long-term hyperglycemic stimulation, corneal endothelial damage may develop independently of diabetic retinopathy. Subclinical morphological abnormalities of the corneal endothelium can be detected in patients without apparent retinal lesions.

The corneal endothelium is a monolayer of nonregenerative hexagonal cells whose barrier and ion-pump functions are essential for maintaining corneal transparency (7, 8). Endothelial cell density (ECD) serves as an indirect marker of endothelial health (9, 10). In children, ECD reaches approximately 4000 cells/mm² (11), declining with age to 2700–2900 cells/mm² in middle-aged adults (12), at an annual physiological loss rate of approximately 0.6% (13, 14). To compensate for cell loss, residual endothelial cells enlarge and migrate, leading to increased polymegathism and pleomorphism (15). When ECD falls below 500–700 cells/mm², endothelial pump function becomes severely impaired, triggering irreversible corneal edema and opacity.17 Currently, corneal transplantation remains the only definitive treatment for irreversible vision loss caused by corneal opacity, imposing substantial financial and psychological burdens on affected patients (16, 17).

Previous studies on the effects of T2DM on corneal endothelial morphology have yielded inconsistent results. Most studies report that T2DM is associated with decreased ECD, increased polymegathism, and reduced hexagonality, with more pronounced changes in patients with prolonged diabetes or DR. However, several others have found no significant differences in endothelial parameters across diabetes duration or DR severity. Despite these conflicting findings, previous meta-analyses have provided preliminary evidence on diabetes-related corneal endothelial changes but were limited by small sample sizes, the inclusion of both type 1 and type 2 diabetes, and the absence of subgroup analyses stratified by DR status and disease duration (18). The present systematic review and meta-analysis updates and extends the existing evidence by incorporating a larger number of more recent studies, restricting the analysis to T2DM exclusively, and introducing three additional subgroup comparisons: DR versus no DR, PDR versus NPDR, and disease duration ≥10 years versus <10 years. By synthesizing the latest global evidence, this study aims to provide a more comprehensive and stratified quantification of corneal endothelial morphological alterations in T2DM, thereby offering evidence-based guidance for preoperative risk assessment and clinical management.

## 2 Methods

### 2.1 Search strategy for study selection

This systematic review adhered to the Preferred Reporting Items for Systematic Reviews and Meta-Analyses (PRISMA) guidelines and the Meta-analysis of Observational Studies in Epidemiology (MOOSE) checklist (**Supplementary File 1** and **Supplementary File 2**) (19, 20). The study protocol was prospectively registered with the International Prospective Register of Systematic Reviews (PROSPERO; Registration ID: CRD420261473313). Two independent investigators (L.Y.R. and L.C.L.) conducted a comprehensive search of five databases (PubMed, Embase, Web of Science, Ovid Medline, and Cochrane Library) from inception through July 15, 2026. The search used a combination of Medical Subject Headings (MeSH) terms and free-text keywords: (“diabetes mellitus, type 2” OR “type 2 diabetes” OR “type 2 DM” OR “T2DM” OR “type II diabetes”) AND (“endothelium, corneal” OR “corneal endothelium” OR “corneal endothelial”). Additional studies were identified through manual searches of reference lists, with full search strategies provided in the **Supplementary Material** (**Supplementary File 3**).

Eligibility criteria were defined according to the PICOS framework (21): Population – patients diagnosed with T2DM and healthy controls; Intervention – not applicable; Comparison – T2DM vs. healthy controls, DR vs. no DR, PDR vs. NPDR and duration ≥10 years vs. <10 years; Outcome – corneal endothelial parameters (ECD, CV and HEX); Study design – cross-sectional studies without language restriction. Exclusion criteria included: (1) animal studies; (2) secondary literature (e.g., reviews, case reports, or letters); (3) studies lacking corneal endothelial cell outcomes; (4) studies with data errors; (5) studies using contact specular microscopy; (6) bilateral-eye studies.

Citavi v5.3 (Swiss Academic Software) was utilized for reference management and duplicate removal. The screening process was carried out in two stages by two independent reviewers (L.Y.R. and L.C.L.): (1) title and abstract screening; (2) full-text assessment against the inclusion and exclusion criteria. Discrepancies were resolved through discussion with a third investigator (L.T.L.).

### 2.2 Data extraction

Data extraction was conducted using a predefined template that captured details such as the first author, publication year, country, race, gender, age, glycated hemoglobin (HbA1c), specular microscopy devices, and endothelial cell parameters. Corneal endothelial parameters were assessed using a non-contact specular microscope. For studies that reported data from both eyes of the same patient, only right eye data were extracted to maintain the independence of observations. For each eye, three images of the central corneal endothelium were captured, and the image with the best quality or the average of the three readings was used for analysis. A minimum of 100 endothelial cells were analyzed per image using either automatic or semi-automatic analysis software. The following parameters were recorded: endothelial cell density (ECD), coefficient of variation in cell size (CV), and percentage of hexagonal cells (HEX). ECD reflects the numerical reserve of the endothelial layer, with lower values indicating reduced cell density. CV represents polymegathism, the variability in individual cell area, and serves as an early and sensitive indicator of endothelial stress. HEX represents pleomorphism, the regularity of cell shape, with a lower percentage of hexagonal cells indicating morphological disruption of the endothelial cells. All definitions strictly followed standardized descriptions from the original studies. Two investigators (L.Y.R. and L.C.L.) independently performed blinded data extraction. Discrepancies were resolved through consensus with a third reviewer (L.T.L.).

### 2.3 Risk of bias assessment

Cross-sectional studies were evaluated using the US Agency for Healthcare Research and Quality (AHRQ) checklist (11 items) (22), which covers data sources, variable definitions, and sample representativeness. Two independent investigators (L.Y.R. and L.C.L.) conducted the risk-of-bias assessments, resolving discrepancies through discussion with a third reviewer (L.T.L.). Studies scoring < 4 on the AHRQ checklist were classified as low quality with a high risk of bias and excluded from the meta-analysis to ensure robustness.

### 2.4 Statistical analysis

Statistical analyses were conducted using Stata v16.0 (Stata Corp). Comparisons included: (1) patients with T2DM vs. healthy controls; (2) DR vs. no DR subgroups; (3) PDR vs. NPDR subgroups; (4) duration ≥10 years vs. <10 years subgroups. Continuous outcomes were expressed as mean ± standard deviation. For studies reporting medians with interquartile ranges (IQR), data were converted to means and standard deviations using the Method for Unknown Non-Normal Distributions (MLN) proposed by Cai et al. (23). For studies presenting data stratified into multiple subgroups, combined group means and standard deviations were derived using the formulas described by Harrer et al. (24, 25). Standardized mean differences (Cohen’s d) with 95% confidence intervals were computed. Cohen’s d was selected because the included studies used different specular microscopy devices with varying automated segmentation algorithms, which may introduce systematic differences in counts despite measuring the same constructs (ECD, CV, and HEX). Heterogeneity was examined using χ² tests and I² statistics: fixed-effect models were adopted when P > 0.10 and I² < 50%; otherwise, random-effects models were employed. Sensitivity analyses (leave-one-out method) were carried out for outcomes with ≥ 3 studies. Publication bias was evaluated using funnel plots and Egger’s test for all outcomes. Statistical significance was set at P < 0.05.

## 3 Results

### 3.1 Literature search results

The database search initially identified 419 articles, comprising 91 from PubMed, 126 from Embase, 176 from Web of Science, 17 from Cochrane, and 9 from Ovid Medline. After removing 86 duplicates, 333 records underwent title and abstract screening. Of these, 292 articles were excluded due to irrelevance to the topic (n = 284) or ineligible publication types (n = 8). The remaining 41 articles were retrieved for full-text assessment. Following full-text review, 20 articles were excluded for the following reasons: presence of other ocular diseases (n = 1), no mean and standard deviation were reported for the endothelial parameters of interest (n = 4), use of contact specular microscopy (n = 7), inclusion of type 1 diabetes patients (n = 2), diabetes type not specified (n = 1), bilateral-eye studies (n = 4), and abnormal and implausible data (n = 1). One additional eligible study was identified from the reference lists of the included articles (26). Consequently, 22 articles were included in both the qualitative synthesis and the meta-analysis. The selection process adhered to PRISMA standards, as illustrated in the flow diagram (**Figure 1**).

**Figure 1:**
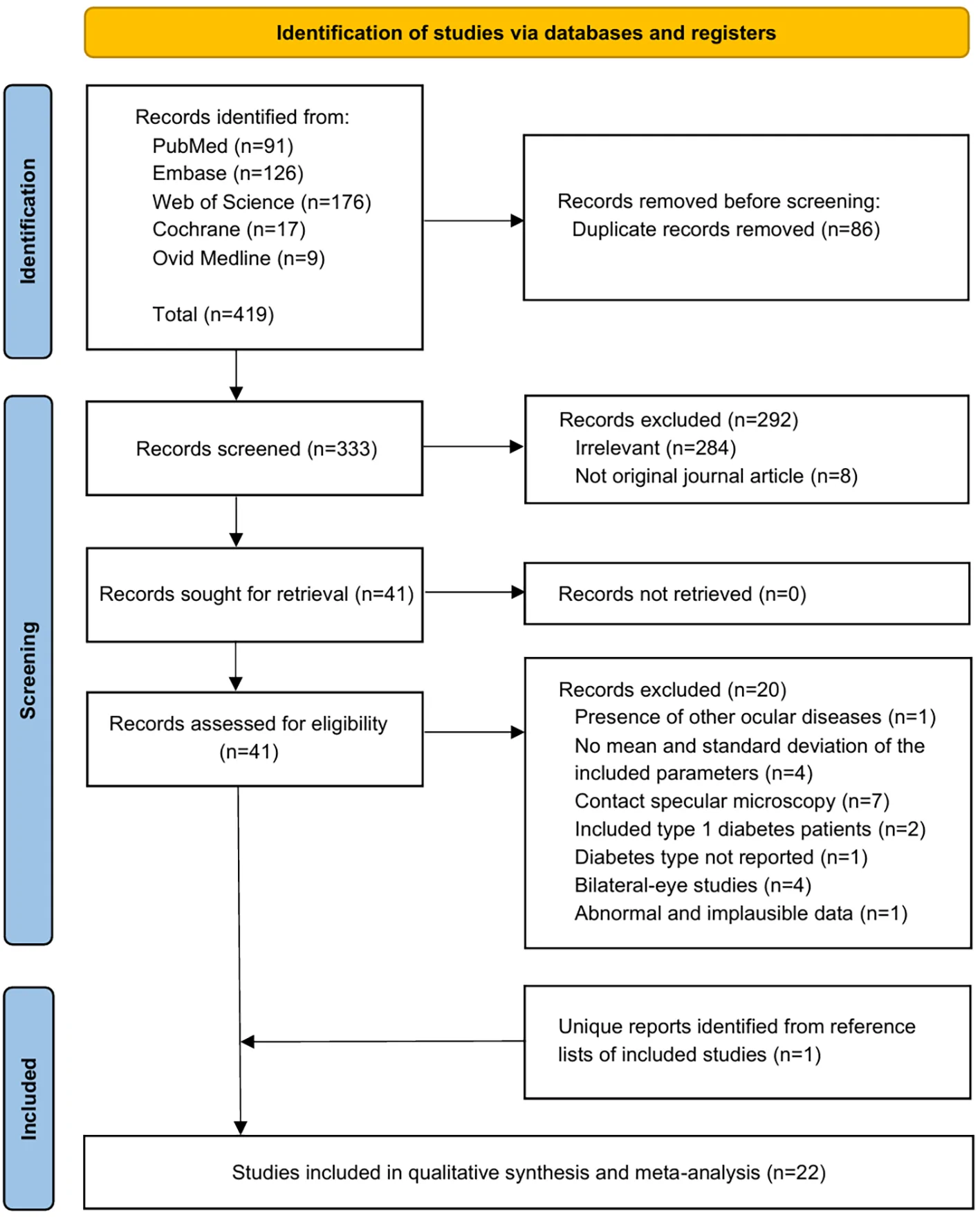
PRISMA Flowchart of selection of studies for inclusion in the meta-analysis.

### 3.2 Primary outcomes and qualitative synthesis

This systematic review included 22 cross-sectional studies (**Table 1**) published between 2002 and 2026, comprising 5 studies from Turkey (27–31), 5 from India (26, 32–35), 2 each from Nepal (36, 37) and Saudi Arabia (38, 39), and 1 each from Poland (40), Iran (41), Austria (42), Portugal (43), Spain (44), Denmark (45), Malaysia (46), and Japan (47). The study populations encompassed Asian, Caucasian, and Middle Eastern or North African participants. The mean patient age ranged from 45.5 to 73.2 years across studies (26–45, 47), though age data were unreported in 1 study (46). Sex distribution was reported in 19 studies (26, 27, 29–32, 34–45, 47), while 3 studies did not provide gender-specific data (28, 33, 46). HbA1c levels were documented in 14 studies (26, 28–34, 38, 40, 43–45, 47), with mean values ranging from 6.77% to 9.2%; the remaining 8 studies did not report HbA1c data (27, 35–37, 39, 41, 42, 46).

**Table 1:** Characteristics of included studies in meta-analysis.

Studies/year	Country	Race	Number of eyes		Gender (M/F)		Age (mean ± SD) or (median [IQR])		HbA1c (%) (mean ± SD)	Specular microscopy devices	Corneal endothelial cell parameters	Analysis groups
			Cases	Controls	Cases	Controls	Cases	Controls				
Aryal 2026 (37)	Nepal	Asian	110	110	64/46	64/46	54 ± 9.93	54 ± 9.93	NA	Nidek CEM-530	ECD, CV, HEX	①②③④
Dhami 2025 (32)	India	Asian	100	100	59/41	46/54	61.22 ± 5.55	60.12 ± 5.28	7.79 ± 1.46	Tomey EM-4000	ECD, CV, HEX	①②③④
Opala 2025 (40)	Poland	Caucasian	80	80	37/43	38/42	72.04 ± 5.60	73.22 ± 4.90	6.77 ± 0.13	Nidek CEM-530	ECD, CV, HEX	①
Sharma 2025 (26)	India	Asian	50	50	31/19	31/19	52.62 ± 10.54	52.62 ± 10.54	7.31 ± 1.13	Nidek CEM-530	ECD, CV, HEX	①
Shrestha 2025 (36)	Nepal	Asian	74	74	35/39	35/39	55.2 ± 10.2	54.5 ± 10.7	NA	Nidek CEM-530	ECD, CV, HEX	①④
Jha 2022 (33)	India	Asian	592	596	NA	NA	62.17 ± 9.49	63.03 ± 11.04	7.5 ± 1.7	Tomey EM-3000	ECD, CV, HEX	①②③④
Mortazavi 2022 (41)	Iran	MENA	134	NA	57/77	NA	61.03 ± 8.08	NA	NA	NA	ECD, CV, HEX	②③
Songur 2022 (27)	Turkey	Caucasian	90	NA	49/41	NA	50.43 ± 12.06	NA	NA	Nidek CEM-530	ECD, CV, HEX	②③
Antičić-Eichwalder 2021 (42)	Austria	Caucasia	Diabetic nonsmokers 50 Diabetic smoker 50	Nondiabetic nonsmokers 50 Nondiabetic smoker 50	Diabetic nonsmokers 16/34 Diabetic smoker 20/30	Nondiabetic nonsmokers 27/23 Nondiabetic smoker 26/24	Diabetic nonsmokers 61.48 (55.15 – 67.81) Diabetic smoker 59.34 (51.98 – 66.70)	Nondiabetic nonsmokers 60.62 (53.80 – 67.44) Nondiabetic smoker 60.72 (54.88 – 66.56)	NA	Topcon SP-3000P	ECD, CV, HEX	①
Chowdhury 2021 (34)	India	Asian	131	131	62/69	62/69	53.26 ± 6.24	53.66 ± 6.73	6.97 ± 0.99	Topcon SP-1P	ECD, CV, HEX	①②③④
Çolak 2020 (28)	Turkey	Caucasian	45	45	NA	NA	53.4 ± 4.2	51.8 ± 4.8	8.2 ± 1.6	Tomey EM-4000	ECD, CV, HEX	①③
Beato 2020 (43)	Portugal	Caucasian	60	47	22/38	18/29	72 ± 6	70 ± 6	7.0 ± 1.1	Topcon SP-3000P	ECD, CV, HEX	①②④
Taşli 2020 (30)	Turkey	Caucasian	195	100	87/108	37/63	61.38 ± 8.44	59.60 ± 7.98	8.14 ± 1.60	Nidek CEM-530	ECD, CV, HEX	①②③
Toprak 2020 (29)	Turkey	Caucasian	100	92	41/59	43/49	53.3 ± 10.4	53.8 ± 8.8	7.1 ± 1.2	Nidek CEM-530	ECD, CV, HEX	①④
Cankurtaran 2019 (31)	Turkey	Caucasian	153	146	76/77	71/75	54.9 ± 6.6	53.9 ± 7.3	8.7 ± 1.8	Topcon SP-3000P	ECD, CV, HEX	①②③
El-Agamy 2017 (38)	Saudi Arabia	MENA	57	45	27/30	22/23	57.08 ± 8.37	50.80 ± 8.39	8.57 ± 2.09	Tomey EM-3000	ECD, CV, HEX	①②③④
Briggs 2015 (39)	Saudi Arabia	MENA	125	90	76/49	42/48	55.8 ± 12	57.1 ± 11.5	NA	Topcon SP-3000P	ECD	①④
Calvo-Maroto 2014 (44)	Spain	Caucasian	Short-term diabetes 37 Long-term diabetes 40	Controls I 38 Controls II 43	Short-term diabetes 16/21 Long-term diabetes 17/23	Controls I 20/18 Controls II 22/21	Short-term diabetes 45.5 ± 2.5 Long-term diabetes 52.2 ± 1.8	Controls I 45.3 ± 2.1 Controls II 52.3 ± 2.6	Short-term diabetes 7.78 ± 0.66 Long-term diabetes 7.66 ± 0.78	Topcon SP-3000P	ECD	①④
Storr-Paulsen 2014 (45)	Denmark	Caucasian	107	128	51/56	49/79	72.1 ± 11.0	75.6 ± 8.9	7.3 ± 1.1	Topcon SP-2000P	ECD, CV, HEX	①
Sudhir 2012 (35)	India	Asian	1191	120	637/554	58/62	54.8 ± 9.5	51.9 ± 8.7	NA	Konan Noncon Robo Ca SP-8000	ECD, CV, HEX	①
Choo 2010 (46)	Malaysia	Asian	100	100	NA	NA	NA	NA	NA	Topcon SP-3000P	ECD, CV, HEX	①
Inoue 2002 (47)	Japan	Asian	99	97	53/46	52/45	65.5 ± 7.5	67.6 ± 7.3	6.9 ± 1.3	NA	ECD, CV, HEX	①

① Type 2 diabetes mellitus vs. controls, ② Diabetic retinopathy vs. no diabetic retinopathy, ③ Proliferative diabetic retinopathy vs. Non-proliferative diabetic retinopathy, ④ Type 2 diabetes mellitus duration ≥10 years vs. <10 years, HbA1c Glycated hemoglobin, NA Not available, ECD Endothelial cell density, CV Coefficient of variation, HEX Percentage of hexagonality, MENA Middle Eastern or North African.

All studies assessed corneal endothelial parameters using non-contact specular microscopy. The most commonly used devices were the Nidek CEM-530, employed in 7 studies (26, 27, 29, 30, 36, 37, 40), followed by the Topcon SP-3000P in 5 studies (31, 39, 42–44), the Tomey EM-3000/EM-4000 in 4 studies (28, 32, 33, 38). ECD was reported in all 22 studies (26–47), while CV and HEX were reported in 20 studies (26–38, 40–43, 45–47). Comprehensive details are presented in **Table 1**.

### 3.3 Quality assessment

Among the 22 included cross-sectional studies, the AHRQ scores ranged from 4 to 7 points, indicating moderate to high methodological quality (**Table 2**). Two studies achieved the highest score of 7 points, while three studies received the lowest score of 4 points. All studies adequately defined the source of information (item 1), listed inclusion and exclusion criteria (item 2), and explained patient exclusions from analysis (item 7). Common methodological limitations included the absence of consecutive patient enrollment (item 4, unclear in 19 studies), lack of masking of outcome assessors (item 5, unclear in 19 studies), failure to report handling of missing data (item 9, unclear in all studies), and insufficient reporting of patient response rates and data completeness (item 10, unclear in 21 studies). As all studies employed a cross-sectional design, none reported follow-up expectations (item 11).

**Table 2:** Risk of bias assessment of included cross-sectional studies.

Studies	1	2	3	4	5	6	7	8	9	10	11	Score
Aryal 2026 (37)	Y	Y	Y	U	U	U	Y	Y	U	U	N	5
Dhami 2025 (32)	Y	Y	U	U	U	Y	Y	Y	U	U	N	5
Opala 2025 (40)	Y	Y	U	U	U	Y	Y	Y	U	U	N	5
Sharma 2025 (26)	Y	Y	U	U	U	Y	Y	Y	U	U	N	6
Shrestha 2025 (36)	Y	Y	Y	Y	U	Y	Y	Y	U	U	N	7
Jha 2022 (33)	Y	Y	U	U	Y	Y	Y	Y	U	U	N	6
Mortazavi 2022 (41)	Y	Y	Y	U	U	U	Y	Y	U	U	N	5
Songur 2022 (27)	Y	Y	Y	U	U	U	Y	U	U	U	N	4
Antičić-Eichwalder 2021 (42)	Y	Y	Y	U	U	Y	Y	Y	U	U	N	6
Chowdhury 2021 (34)	Y	Y	Y	Y	U	U	Y	Y	U	U	N	6
Çolak 2020 (28)	Y	Y	Y	U	U	U	Y	U	U	U	N	4
Beato 2020 (43)	Y	Y	Y	U	U	Y	Y	Y	U	U	N	6
Taşli 2020 (30)	Y	Y	Y	U	U	U	Y	Y	U	U	N	5
Toprak 2020 (29)	Y	Y	U	U	U	U	Y	Y	U	U	N	4
Cankurtaran 2019 (31)	Y	Y	U	U	Y	Y	Y	Y	U	U	N	6
El-Agamy 2017 (38)	Y	Y	Y	U	U	Y	Y	N	U	U	N	5
Briggs 2015 (39)	Y	Y	Y	U	U	U	Y	Y	U	U	N	5
Calvo-Maroto 2014 (44)	Y	Y	U	U	U	Y	Y	Y	U	U	N	6
Storr-Paulsen 2014 (45)	Y	Y	U	U	Y	Y	Y	Y	U	U	N	6
Sudhir 2012 (35)	Y	Y	U	Y	U	U	Y	Y	U	Y	N	7
Choo 2010 (46)	Y	Y	Y	U	U	Y	Y	U	U	U	N	5
Inoue 2002 (47)	Y	Y	Y	U	U	Y	Y	Y	U	U	N	6

Y yes, N no, U unclear.

US Agency for Healthcare Research and Quality, AHRQ: 1 = Define the source of information (survey, record review); 2 = List inclusion and exclusion criteria for exposed and unexposed subjects (cases and controls) or refer to previous publications; 3 = Indicate time period used for identifying patients; 4 = Indicate whether or not subjects were consecutive if not population-based; 5 = Indicate if evaluators of subjective components of study were masked to other aspects of the status of the participants; 6 = Describe any assessments undertaken for quality assurance purposes (e.g., test/retest of primary outcome measurements); 7 = Explain any patient exclusions from analysis; 8 = Describe how confounding was assessed and/or controlled; 9 = If applicable, explain how missing data were handled in the analysis; 10 = Summarize patient response rates and completeness of data collection; 11 = Clarify what follow-up, if any, was expected and the percentage of patients for which incomplete data or follow-up was obtained.

Publication bias was evaluated using funnel plots and Egger’s test across all meta-analysis outcomes. Funnel plots appeared asymmetric for three outcomes: CV in the T2DM vs. control comparison, ECD in the PDR vs. NPDR comparison, and ECD in the duration ≥10 years vs. <10 years comparison. Egger’s test revealed significant publication bias CV in the duration ≥10 years vs. <10 years comparison (P = 0.010). For the remaining outcomes, Egger’s test was nonsignificant (all P > 0.05), and funnel plots were generally symmetric, suggesting a low likelihood of publication bias (**Supplementary File 4**).

Trim-and-fill analysis was performed for the five outcomes with suspected publication bias. After imputation of missing studies, the pooled estimates remained largely unchanged for four outcomes, confirming the robustness of the primary findings. However, for CV in the duration ≥10 years vs. <10 years comparison, the trim-and-fill analysis yielded a reversed result, indicating that this finding was not robust and should be interpreted with caution (**Supplementary File 5**).

### 3.4 Results of meta-analysis

#### 3.4.1 Corneal endothelial parameters in patients with T2DM and healthy controls

Twenty studies (26, 28–40, 42–47) involving 5,878 eyes (**Figure 2**; **Table 3**) showed significantly lower ECD in patients with T2DM compared with healthy controls (Cohen’s d = −0.35, 95% CI −0.49 to −0.21; P < 0.001), with substantial heterogeneity (I² = 82%). Eighteen studies (26, 28–38, 40, 42, 43, 45–47) involving 5,505 eyes demonstrated significantly higher CV in the T2DM group (Cohen’s d = 0.25, 95% CI 0.16 to 0.33; P < 0.001), with moderate heterogeneity (I² = 46%). Eighteen studies (26, 28–38, 40, 42, 43, 45–47) involving 5,505 eyes showed significantly lower HEX in patients with T2DM (Cohen’s d = −0.28, 95% CI −0.47 to −0.09; P < 0.001), with substantial heterogeneity (I² = 90%). Leave-one-out sensitivity analysis confirmed the robustness of all pooled estimates (**Supplementary File 6**).

**Figure 2:**
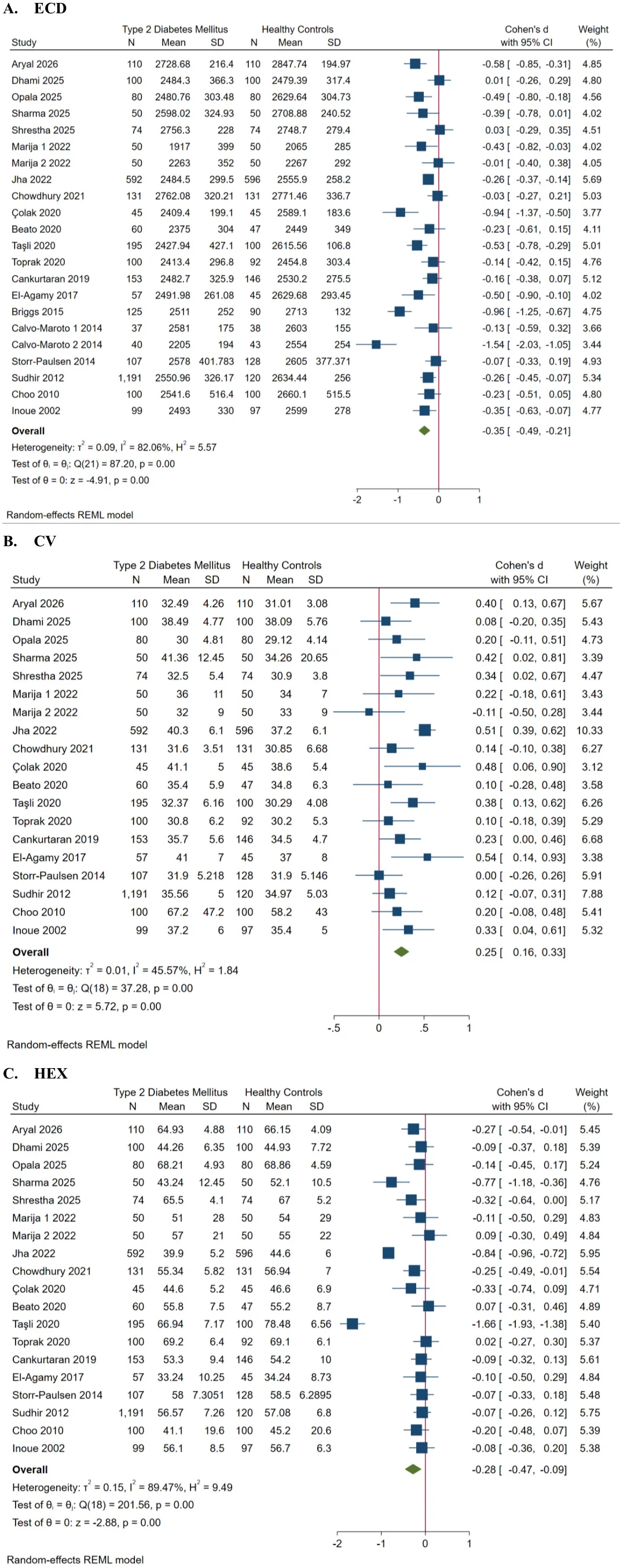
Forest plot of Cohen's d in corneal endothelial parameters between type 2 diabetes mellitus (T2DM) and healthy controls. **(A)** ECD (endothelial cell density); **(B)** CV (coefficient of variation); **(C)** HEX (percentage of hexagonality).

**Table 3:** Meta-analysis summary of corneal endothelial parameters in type 2 diabetes mellitus.

Parameters	Number of studies	Eyes	Cohen’s d	95%CI	P-value	I^2^
T2DM vs. Controls						
ECD	20	5,878	−0.35	[−0.49, −0.21]	<0.001^***^	82%
CV	18	5,505	0.25	[0.16, 0.33]	<0.001^***^	46%
HEX	18	5,505	−0.28	[−0.47, −0.09]	<0.001^***^	90%
DR vs. no DR						
ECD	10	1,622	−0.22	[−0.36, −0.08]	0.002^**^	34%
CV	10	1,622	0.26	[0.08, 0.45]	0.005^**^	63%
HEX	10	1,622	−0.23	[−0.42, −0.04]	0.02^*^	65%
PDR vs. NPDR						
ECD	10	907	−0.21	[−0.49, 0.07]	0.14	70%
CV	10	907	0.21	[0.00, 0.41]	0.05	43%
HEX	10	907	−0.06	[−0.21, 0.08]	0.37	16%
Duration ≥10 years vs. <10 years						
ECD	10	1,426	−0.39	[−0.75, −0.02]	0.04^*^	89%
CV	8	1,224	0.15	[−0.03, 0.33]	0.10	44%
HEX	8	1,224	−0.18	[−0.29, −0.06]	0.003^**^	0%

^*^ indicates P < 0.05, ^**^ indicates P < 0.01, ^***^ indicates P < 0.001;.

#### 3.4.2 Corneal endothelial parameters in eyes with DR and without DR

Ten studies (27, 30–34, 37, 38, 41, 43) involving 1,622 eyes (**Figure 3** and **Table 3**) showed significantly lower ECD in eyes with DR compared with eyes without DR (Cohen’s d = −0.22, 95% CI −0.36 to −0.08; P = 0.002), with low heterogeneity (I² = 34%). The same ten studies (27, 30–34, 37, 38, 41, 43) demonstrated significantly higher CV in the DR group (Cohen’s d = 0.26, 95% CI 0.08 to 0.45; P = 0.005), with moderate heterogeneity (I² = 63%), and significantly lower HEX (Cohen’s d = −0.23, 95% CI −0.42 to −0.04; P = 0.02), with moderate heterogeneity (I² = 65%). Leave-one-out sensitivity analysis confirmed the robustness of all pooled estimates (**Supplementary File 6**).

**Figure 3:**
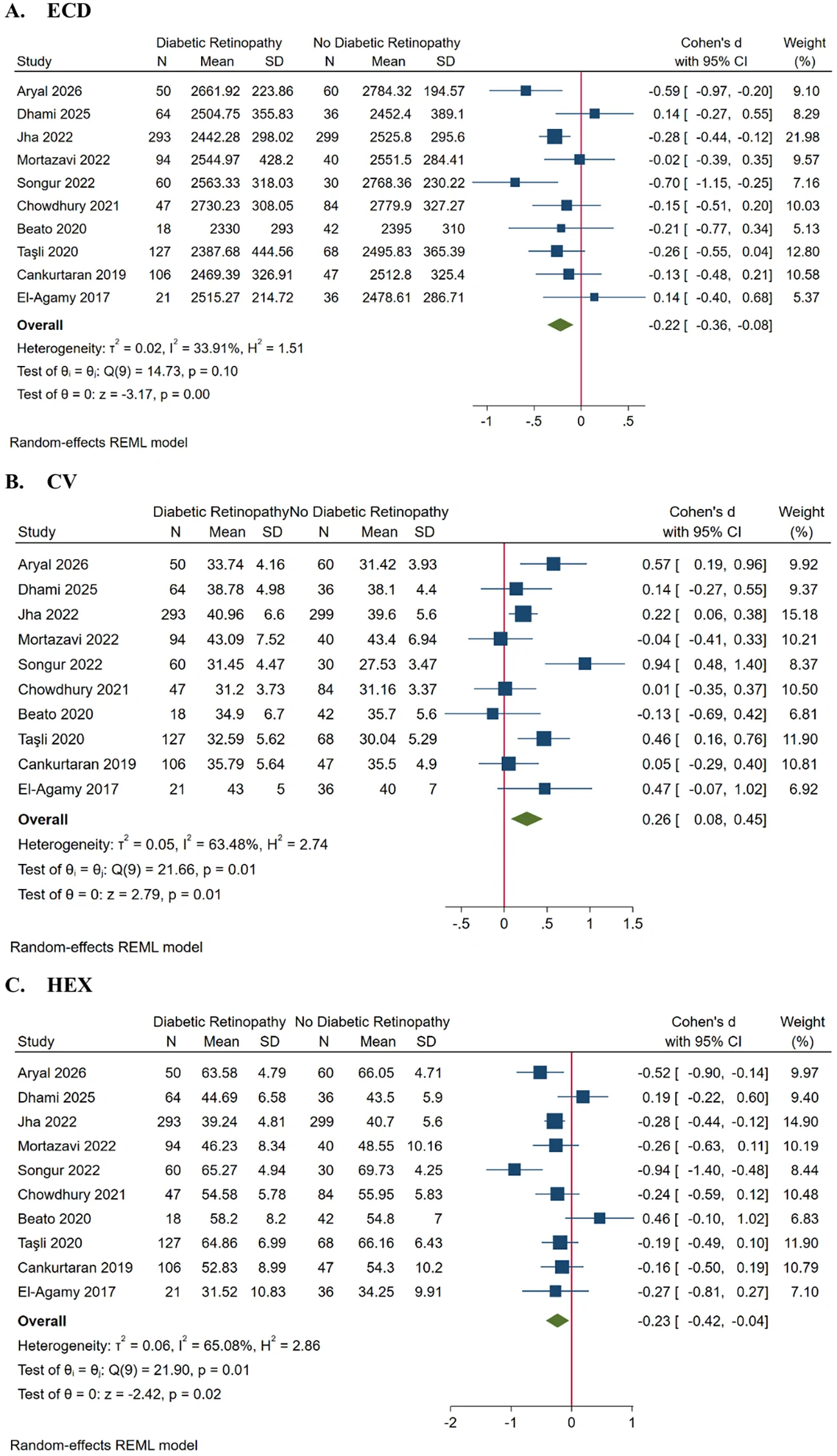
Forest plot of Cohen's d in corneal endothelial parameters between between diabetic retinopathy (DR) and no diabetic retinopathy groups. **(A)** ECD (endothelial cell density); **(B)** CV (coefficient of variation); **(C)** HEX (percentage of hexagonality).

#### 3.4.3 Corneal endothelial parameters in PDR and NPDR

Ten studies (27, 28, 30–34, 37, 38, 41) involving 907 eyes (**Figure 4**, **Table 3**) were included in the PDR versus NPDR comparison. No significant differences were observed for ECD (Cohen’s d = −0.21, 95% CI −0.49 to 0.07; P = 0.14; I² = 70%), CV (Cohen’s d = 0.21, 95% CI 0.00 to 0.41; P = 0.05; I² = 43%) or HEX (Cohen’s d = −0.06, 95% CI −0.21 to 0.08; P = 0.37; I² = 16%). Leave-one-out sensitivity analysis confirmed the robustness of the pooled estimates for ECD and HEX. However, for CV, the result became nonsignificant upon the exclusion of Aryal 2026 (37), Jha 2022 (33), Mortazavi 2022 (41), Songur 2022 (27), Chowdhury 2021 (34), Çolak 2020 (28), or Taşlı 2020 (30), suggesting that this finding was not robust and should be interpreted with caution (**Supplementary File 6**).

**Figure 4:**
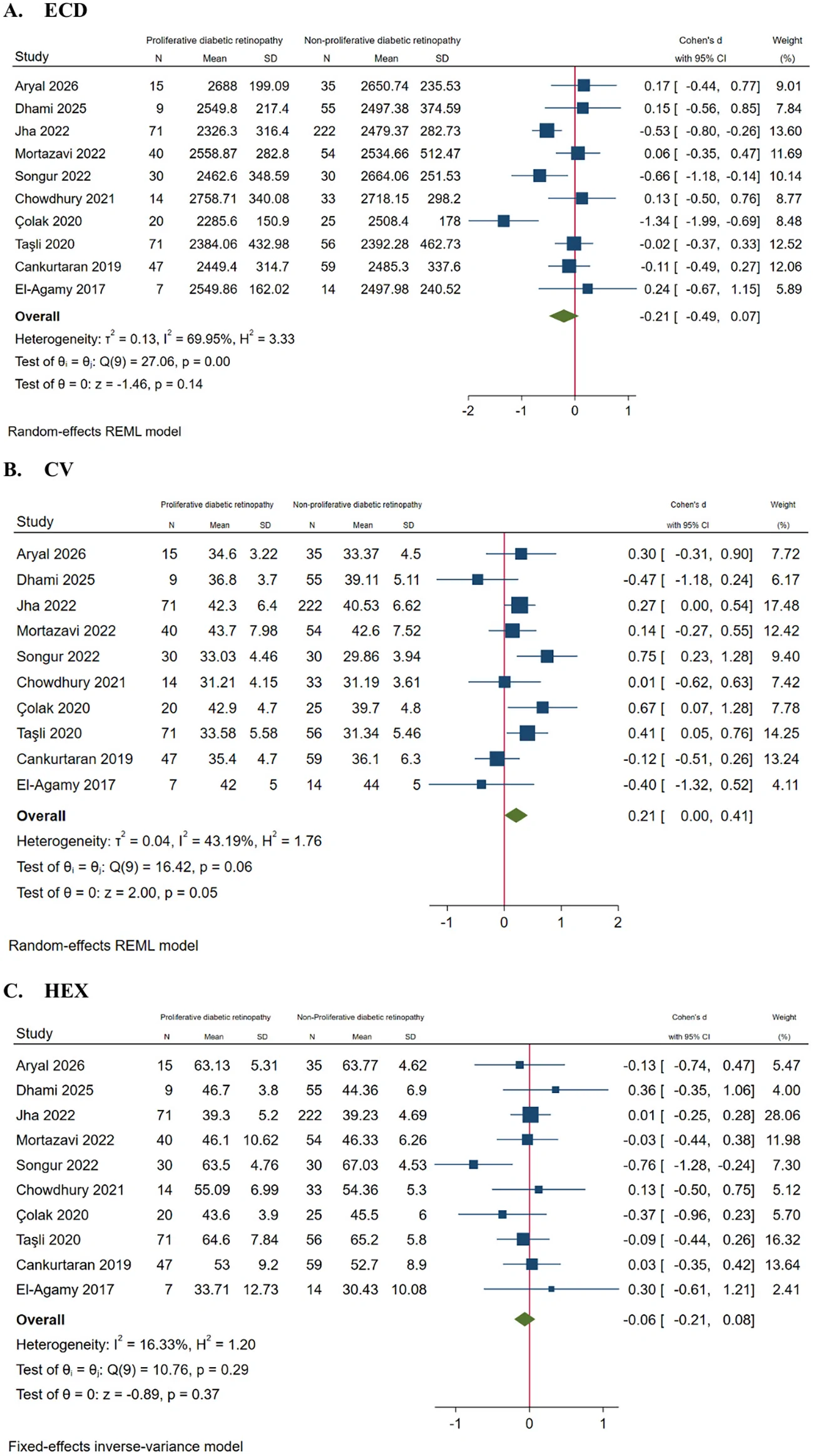
Forest plot of Cohen's d in corneal endothelial parameters between proliferative diabetic retinopathy (PDR) and non-proliferative diabetic retinopathy (NPDR) groups. **(A)**ECD (endothelial cell density); **(B)** CV (coefficient of variation); **(C)** HEX (percentage of hexagonality).

#### 3.4.4 Corneal endothelial parameters by disease duration ≥10 years and <10 years

Ten studies (29, 32–34, 36–39, 43, 44) involving 1,426 eyes (**Figure 5**; **Table 3**) showed significantly cduration <10 years (Cohen’s d = −0.39, 95% CI −0.75 to −0.02; P = 0.04), with substantial heterogeneity (I² = 89%). Eight studies (29, 32–34, 36–38, 43) involving 1,224 eyes demonstrated no significant difference in CV between the two duration groups (Cohen’s d = 0.15, 95% CI −0.03 to 0.33; P = 0.10; I² = 44%). Eight studies (29, 32–34, 36–38, 43) involving 1,224 eyes showed significantly lower HEX in the longer duration group (Cohen’s d = −0.18, 95% CI −0.29 to −0.06; P = 0.003), with no heterogeneity (I² = 0%).

**Figure 5:**
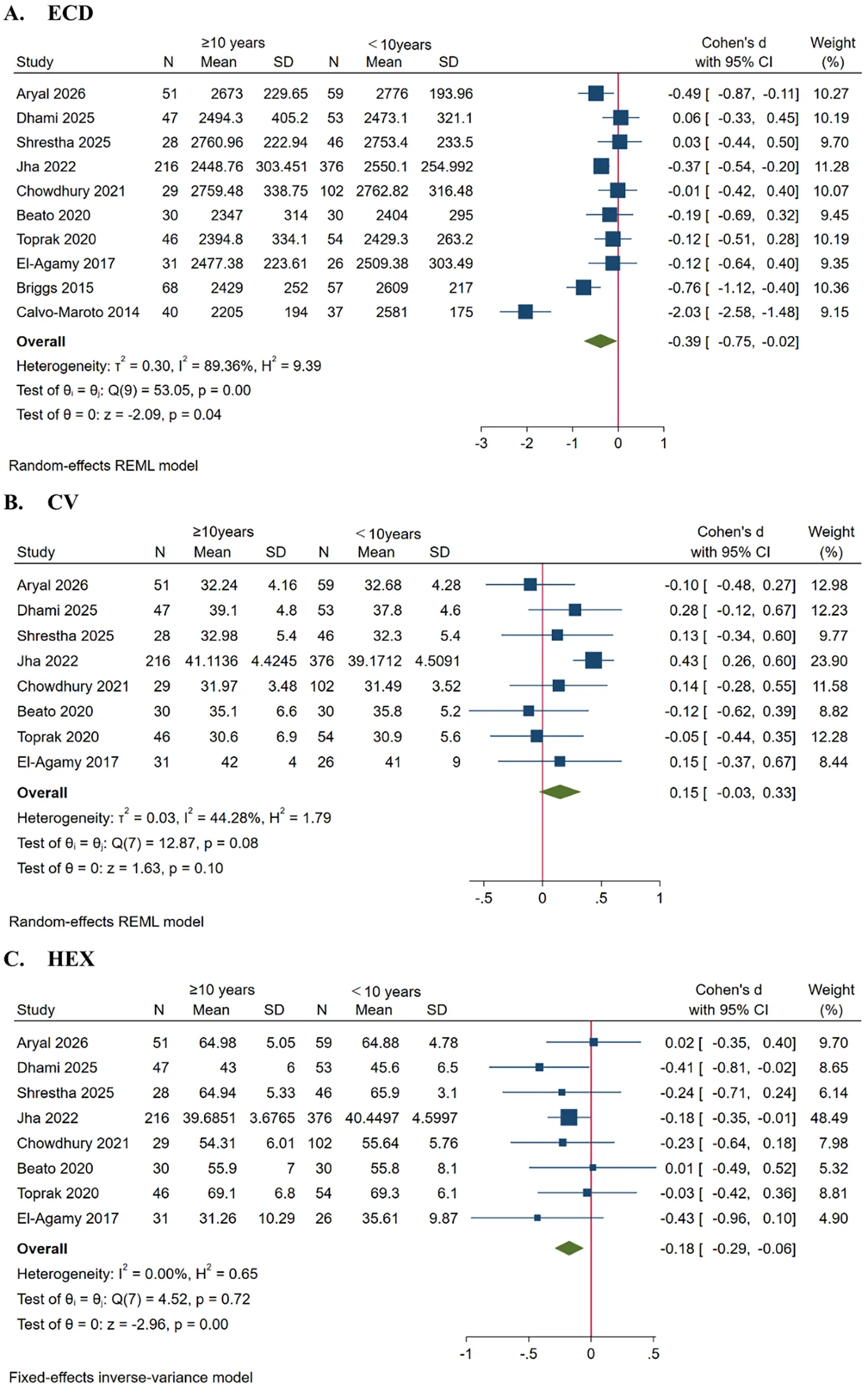
Forest plot of Cohen's d in corneal endothelial parameters between patients with type 2 diabetes mellitus (T2DM) duration ≥10 years and <10 years groups. **(A)** ECD (endothelial cell density); **(B)** CV (coefficient of variation); **(C)** HEX (percentage of hexagonality).

Leave-one-out sensitivity analysis confirmed the robustness of the pooled estimates for ECD and HEX. However, for CV, the nonsignificant result became significant upon the exclusion of Aryal 2026 (37), suggesting that this finding was sensitive to the influence of a single study (**Supplementary File 6**). Furthermore, trim-and-fill analysis for CV in this comparison yielded a reversed result after imputation of missing studies, indicating that this finding was not robust and should be interpreted with caution (**Supplementary File 5**).

## 4 Discussion

This systematic review and meta-analysis synthesized data from 22 cross-sectional studies involving 5,878 eyes to evaluate corneal endothelial changes in T2DM across four comparisons: T2DM versus healthy controls, DR versus no DR, PDR versus NPDR, and disease duration ≥10 years versus <10 years. The results suggest that T2DM is associated with lower ECD and HEX and higher CV, with more pronounced changes in the presence of DR and longer disease duration. However, several pooled estimates appeared sensitive to individual study influences or potential publication bias, warranting cautious interpretation. All included studies employed a cross-sectional design; therefore, the observed associations reflect group-level differences at a single time point and should not be interpreted as evidence of longitudinal progression or causal relationships.

The main T2DM-control analysis included 5,878 eyes. T2DM was associated with lower ECD (Cohen’s d = −0.35), higher CV (Cohen’s d = 0.25), and lower HEX (Cohen’s d = −0.28). These findings are broadly consistent with most primary studies (37–39, 47, 48) and align with two earlier meta-analyses that identified the same overall pattern (18, 49). The reduction in ECD may indicate a loss of endothelial reserve, while the increase in CV and decrease in HEX likely reflect progressive polymegathism and pleomorphism, which are early morphological signs of endothelial stress. These changes could result from chronic hyperglycemia, which promotes the accumulation of advanced glycation end products, sorbitocl-driven osmotic stress, and oxidative damage to endothelial cells.

All three overall estimates remained significant in every leave-one-out analysis. Heterogeneity was nevertheless substantial for HEX (I² = 90%). This high heterogeneity may reflect differences in image quality, cell-count thresholds, device-specific algorithms, and the ethnic diversity of study populations. Egger tests for overall CV (P = 0.056) and HEX (P = 0.057) approached but did not reach the conventional significance threshold. Trim-and-fill did not alter the CV estimate, whereas the HEX analysis imputed five hypothetical missing studies and retained a significant negative estimate (Cohen’s d = −0.379, 95% CI −0.548 to −0.210). The direction of the HEX association appears more consistent than its magnitude, and these microscopy findings indicate structural alteration but do not by themselves prove impaired endothelial pump function or clinical decompensation. Individual studies have reported mixed findings: Schultz et al. (48) found increased CV and reduced HEX without lower ECD, later studies reported lower ECD and less favorable morphology in some populations (50–52), and others found smaller or nonsignificant differences (26, 45). Variation in sample size, population characteristics, glycemic exposure, and measurement protocols may partly explain these discrepancies.

Among 1,622 eyes, those with DR had lower ECD (Cohen’s d = −0.22), higher CV (Cohen’s d = 0.26), and lower HEX (Cohen’s d = −0.23) than those without DR. ECD and CV remained significant in every leave-one-out analysis. The HEX estimate was less stable, becoming borderline after exclusion of Aryal 2026 (37) (P = 0.050) or Jha 2022 (33) (P = 0.056). These findings suggest that eyes with DR tend to show more pronounced corneal endothelial alterations compared with eyes without DR. However, because all data were cross-sectional, this pattern should not be interpreted as evidence that endothelial damage progresses with advancing DR. Dhami et al. (32) found no significant differences across DR grades, whereas Jha et al. (33) reported progressively adverse endothelial changes with increasing DR severity, and Mortazavi et al. (41) found no significant difference between eyes with and without DR. The pooled data nevertheless indicate average group differences across all three indices. DR may mark greater cumulative glycemic, microvascular, and systemic disease burden, but it should not be interpreted as the direct cause of the corneal changes. Age, HbA1c, renal disease, hypertension, and other microvascular complications were not consistently controlled across studies (53). From a clinical perspective, these results suggest that preoperative specular microscopy may be beneficial in diabetic patients with DR to assess endothelial reserve before intraocular surgery (54, 55).

The evidence did not show a clear additional severity gradient between PDR and NPDR. Neither ECD (Cohen’s d = −0.21; P = 0.14) nor HEX (Cohen’s d = −0.06; P = 0.373) differed significantly. The CV estimate was borderline (Cohen’s d = 0.21; P = 0.045) and became nonsignificant when several individual studies were removed. The absence of clear differences could be attributable to several factors. Interpretation is limited by the total sample of 907 eyes and small PDR strata in some studies. Several studies excluded eyes with previous retinal photocoagulation or intravitreal therapy, potentially underrepresenting treated PDR (32, 33). Differences in DR grading, disease duration, and glycemic exposure may have further weakened the contrast between stages. Corneal endothelial damage may also plateau at advanced DR stages, with the most pronounced changes occurring during the transition from no DR to early DR rather than between NPDR and PDR (56).

Associations with disease duration differed by outcome. A duration of at least 10 years was associated with lower ECD (Cohen’s d = −0.39) and lower HEX (Cohen’s d = −0.18). The HEX estimate remained significant in all eight leave-one-out analyses and showed no observed heterogeneity (I² = 0%), which is consistent with the hypothesis that prolonged glycemic exposure may be associated with greater endothelial cell loss. By contrast, the ECD estimate was highly heterogeneous (I² = 89%) and became nonsignificant after exclusion of Aryal 2026 (37), Jha 2022 (33), or Briggs 2015 (39), and should therefore be interpreted cautiously. The high heterogeneity and sensitivity to individual study exclusion further limit the generalizability of this estimate. Individual studies reported different duration-related patterns, with some finding lower ECD and others finding clearer changes in CV or morphology (28, 35, 44, 57). Calvo-Maroto et al. (44) compared a newly diagnosed group with a long-standing diabetes group, creating a wider separation than studies based on a simple 10-year dichotomy. The 10-year cutoff was selected because it is the most commonly reported duration threshold in the primary literature and has been used in previous meta-analyses on diabetes-related ocular complications. This cutoff also approximates the median disease duration in most included studies and provides a clinically meaningful distinction between early-stage and long-standing T2DM. The 10-year cutoff is therefore a pragmatic study threshold rather than a biological breakpoint, and duration since diagnosis may underestimate true glycemic exposure because T2DM can be present before clinical recognition. Evidence for an association between duration and CV was less stable. The primary estimate was nonsignificant (Cohen’s d = 0.15, 95% CI −0.03 to 0.33), but became significant after exclusion of Aryal 2026 (37) and after trim-and-fill imputation (Cohen’s d = 0.312, 95% CI 0.126 to 0.497; Egger P = 0.010). Only eight estimates were available, limiting the reliability of both Egger regression and trim-and-fill. These analyses prove neither an association nor publication bias, leaving the duration-CV relationship uncertain.

Several mechanisms could plausibly contribute to the observed structural changes, although none was measured directly in the included studies. Native endothelial cells from human donor eyes with advanced diabetes showed reduced mitochondrial respiratory capacity and abnormal mitochondrial structure (58). In high-glucose models, impaired mitophagy was accompanied by lower expression of pump and barrier proteins, including Na^+^/K^+^-ATPase and ZO-1 (55). Glycation of extracellular matrix proteins in Descemet’s membrane may impair endothelial adhesion and spreading (59), and aldose reductase inhibition in diabetic animals suggests a possible role for the polyol pathway (60). Other work points to impaired antioxidant defense, increased oxidative susceptibility, and inflammatory cell death signaling (61, 62). These mechanisms offer plausible routes through which chronic metabolic stress could reduce endothelial resilience, but they do not identify the cause of the pooled differences. Because adult human endothelial cells have limited ability to divide, surviving cells generally enlarge and spread in response to cell loss or stress, which is consistent with higher CV and lower HEX (15, 63).

This meta-analysis has several strengths. It represents one of the largest syntheses to date, incorporating 22 studies with 5,878 eyes, substantially exceeding previous meta-analyses. Restricting the analysis to T2DM exclusively reduces the potential confounding from mixing type 1 and type 2 diabetes. The three additional subgroup comparisons offer a more detailed perspective on how corneal endothelial parameters vary across different retinopathy statuses and durations of glycemic exposure. The protocol was prospectively registered, five databases were searched without language restriction, and study selection, data extraction, and risk-of-bias assessment were conducted independently. Restricting inclusion to non-contact specular microscopy reduced measurement heterogeneity, while extracting only right eye data preserved independence of observations. Prespecified comparisons were complemented by leave-one-out analyses, funnel plots, Egger’s tests, and trim-and-fill analyses.

Several limitations should be acknowledged. All included studies were cross-sectional, precluding causal inference. The AHRQ quality assessment revealed common weaknesses: consecutive enrollment was unclear in 19 of 22 studies, masking of assessors was unclear in 19 studies, missing data handling was unreported in all studies, and response rates were reported in only one study. Substantial heterogeneity was observed for several outcomes. Differences in population characteristics, device model, image selection, DR definitions, and glycemic control may have contributed, but incomplete reporting prevented informative meta-regression. Several pooled estimates were not robust in sensitivity analyses or showed signs of possible publication bias, particularly for HEX and CV outcomes. Converting medians with interquartile ranges to means with standard deviations, and combining stratified subgroups, introduced additional approximation. Several subgroup analyses contained only 8–10 estimates, limiting precision. The exclusion of contact specular microscopy studies and bilateral-eye studies, while methodologically justified, may have reduced eligible studies. No formal certainty-of-evidence framework was applied. The findings apply only to T2DM and should not be extrapolated to type 1 diabetes. The use of Cohen’s d rather than raw mean differences was necessary because endothelial parameters were obtained from different specular microscope models with varying image analysis algorithms, which may produce systematic offsets in absolute values. While this approach standardizes the effect sizes and facilitates comparison, it limits the direct clinical interpretability of the pooled estimates in absolute units. Age, HbA1c, hypertension, renal function, and DR severity were inconsistently or not reported and could not be examined as moderators. The observed endothelial changes may therefore partly reflect these systemic factors rather than diabetes alone.

These findings are better interpreted as markers of risk than as clinical thresholds. Specular microscopy characterizes endothelial density and morphology but does not directly measure pump function or individual functional reserve (64). Diabetic corneas may nevertheless be more susceptible to operative stress: a prospective study found greater endothelial cell loss after phacoemulsification in T2DM patients, and a subsequent meta-analysis reported lower postoperative ECD and slower morphological recovery in diabetic eyes (65, 66). The reduction in ECD and increase in CV in T2DM patients, even without DR, raise the possibility that subclinical endothelial damage could precede retinal manifestations. These group-level morphological differences do not establish a specific ECD threshold for surgical risk. Preoperative specular microscopy may be considered on an individualized basis, particularly in patients with DR or disease duration ≥10 years. Patients with DR or disease duration ≥10 years may be at higher risk of endothelial decompensation and could benefit from protective surgical measures, such as dispersive viscoelastic agents and minimized phacoemulsification energy. The available evidence does not establish a universal screening rule or a specific ECD threshold.

Future studies could employ prospective longitudinal designs to help establish temporal relationships between glycemic control, DR progression, and corneal endothelial deterioration. Standardized DR grading and consistent reporting of HbA1c, diabetes duration, and specular microscopy protocols would facilitate more robust studies. Studies investigating the impact of glycemic control interventions would help clarify whether optimizing HbA1c can slow or reverse endothelial damage. Continuous measures of disease duration and glycemic exposure, rather than dichotomized cutoffs, may better capture cumulative metabolic burden. Automated, operator-independent specular microscopy software may reduce measurement variability and improve the reliability of endothelial assessment in both research and clinical settings.

## Data Availability

The original contributions presented in the study are included in the article/**Supplementary Material**. Further inquiries can be directed to the corresponding author.
