# Supplementary material for: Corneal endothelial changes in type 2 diabetes mellitus: a systematic review and meta-analysis with subgroup comparisons by diabetic retinopathy status and disease duration
Source: Front Endocrinol (Lausanne). 2026 Sep 29;17:1982467. doi: 10.3389/fendo.2026.1982467 (PMC13657926; doi:10.3389/fendo.2026.1982467)
Supplement: Supplementary file 3 [file SupplementaryFile3.docx]

| **PubMed** From inception to July 15th, 2026 | | |
| --- | --- | --- |
| #1 | ("diabetes mellitus, type 2"[MeSH Terms] OR "type 2 diabetes"[Title/Abstract] OR "type 2 DM"[Title/Abstract] OR "T2DM"[Title/Abstract] OR "type II diabetes"[Title/Abstract]) AND ("endothelium, corneal"[MeSH Terms] OR "corneal endothelium"[Title/Abstract] OR "corneal endothelial"[Title/Abstract]) | **91** |
| **Web of science** From inception to July 15th, 2026 | | |
| #1 | ((((((((((AB=(diabetes mellitus, type 2)) OR TI=(diabetes mellitus, type 2)) OR AB=(type 2 diabetes)) OR TI=(type 2 diabetes)) OR AB=(type 2 DM)) OR TI=(type 2 DM)) OR AB=(T2DM)) OR TI=(T2DM)) OR AB=(type II diabetes)) OR TI=(type II diabetes)) | **401927** |
| #2 | ((((((AB=(endothelium, corneal)) OR TI=(endothelium, corneal)) OR AB=(corneal endothelium)) OR TI=(corneal endothelium)) OR AB=(corneal endothelial)) OR TI=(corneal endothelial)) | **22776** |
| #3 | #1 AND #2 | **176** |
| **Cochrane** From inception to July 15th, 2026 | | |
| #1 | MeSH descriptor: [diabetes mellitus, type 2] explode all trees | **28060** |
| #2 | (type 2 diabetes):ti,ab,kw | **70241** |
| #3 | (type 2 DM):ti,ab,kw | **3278** |
| #4 | (T2DM):ti,ab,kw | **10533** |
| #5 | (type II diabetes):ti,ab,kw | **7019** |
| #6 | #1 or #2 or #3 or #4 or #5 | **72336** |
| #7 | MeSH descriptor: [endothelium, corneal] explode all trees | **493** |
| #8 | (corneal endothelium):ti,ab,kw | **1009** |
| #9 | (corneal endothelial):ti,ab,kw | **1667** |
| #10 | #7 or #8 or #9 | **1793** |
| #11 | #6 and #10 | **17** |
| **Embase** From inception to July 15th, 2026 | | |
| #1 | 'diabetes mellitus, type 2'/exp OR 'type 2 diabetes':ab,ti OR 'type 2 dm':ab,ti OR 't2dm':ab,ti OR 'type ii diabetes':ab,ti | **529671** |
| #2 | 'endothelium, corneal'/exp OR 'corneal endothelium':ab,ti OR 'corneal endothelial':ab,ti | **14801** |
| #3 | #1 AND #2 | **126** |
| **Medline** From inception to July 15th, 2026 | | |
| #1 | Tl diabetes mellitus, type 2 OR AB diabetes mellitus, type 2 OR Tl type 2 diabetes OR AB type 2 diabetes OR Tl type 2 DM OR AB type 2 DM OR Tl T2DM OR AB T2DM OR Tl type II diabetes OR AB type II diabetes | **74467** |
| #2 | Tl endothelium, corneal OR AB endothelium, corneal OR TI corneal endothelium OR AB corneal endothelium OR Tl corneal endothelial OR AB corneal endothelial | **723** |
| #3 | S1 OR S2 | **9** |
