# Supplementary material for: Corneal endothelial changes in type 2 diabetes mellitus: a systematic review and meta-analysis with subgroup comparisons by diabetic retinopathy status and disease duration
Source: Front Endocrinol (Lausanne). 2026 Sep 29;17:1982467. doi: 10.3389/fendo.2026.1982467 (PMC13657926; doi:10.3389/fendo.2026.1982467)
Supplement: Supplementary file 4 [file SupplementaryFile4.docx]

**Supplementary File 4**

Figure 1: Funnel plot of endothelial cell density (ECD) between type 2 diabetes mellitus (T2DM) and healthy controls

Figure 2: Egger’s test of endothelial cell density (ECD) between type 2 diabetes mellitus (T2DM) and healthy controls

Figure 3: Funnel plot of coefficient of variation (CV) between type 2 diabetes mellitus (T2DM) and healthy controls

Figure 4: Egger’s test of coefficient of variation (CV) between type 2 diabetes mellitus (T2DM) and healthy controls

Figure 5: Funnel plot of percentage of hexagonality (HEX) between type 2 diabetes mellitus (T2DM) and healthy controls

Figure 6: Egger’s test of percentage of hexagonality (HEX) between type 2 diabetes mellitus (T2DM) and healthy controls

Figure 7: Funnel plot of endothelial cell density (ECD) between diabetic retinopathy (DR) and no diabetic retinopathy groups

Figure 8: Egger’s test of endothelial cell density (ECD) between diabetic retinopathy (DR) and no diabetic retinopathy groups

Figure 9: Funnel plot of coefficient of variation (CV) between diabetic retinopathy (DR) and no diabetic retinopathy groups

Figure 10: Egger’s test of coefficient of variation (CV) between diabetic retinopathy (DR) and no diabetic retinopathy groups

Figure 11: Funnel plot of percentage of hexagonality (HEX) between diabetic retinopathy (DR) and no diabetic retinopathy groups

Figure 12: Egger’s test of percentage of hexagonality (HEX) between diabetic retinopathy (DR) and no diabetic retinopathy groups

Figure 13: Funnel plot of endothelial cell density (ECD) between proliferative diabetic retinopathy (PDR) and non-proliferative diabetic retinopathy (NPDR) groups

Figure 14: Egger’s test of endothelial cell density (ECD) between proliferative diabetic retinopathy (PDR) and non-proliferative diabetic retinopathy (NPDR) groups

Figure 15: Funnel plot of coefficient of variation (CV) between proliferative diabetic retinopathy (PDR) and non-proliferative diabetic retinopathy (NPDR) groups

Figure 16: Egger’s test of coefficient of variation (CV) between proliferative diabetic retinopathy (PDR) and non-proliferative diabetic retinopathy (NPDR) groups

Figure 17: Funnel plot of percentage of hexagonality (HEX) between proliferative diabetic retinopathy (PDR) and non-proliferative diabetic retinopathy (NPDR) groups

Figure 18: Egger’s test of percentage of hexagonality (HEX) between proliferative diabetic retinopathy (PDR) and non-proliferative diabetic retinopathy (NPDR) groups

Figure 19: Funnel plot of endothelial cell density (ECD) between patients with type 2 diabetes mellitus (T2DM) duration ≥10 years and <10 years groups

Figure 20: Egger’s test of endothelial cell density (ECD) between patients with type 2 diabetes mellitus (T2DM) duration ≥10 years and <10 years groups

Figure 21: Funnel plot of coefficient of variation (CV) between patients with type 2 diabetes mellitus (T2DM) duration ≥10 years and <10 years groups

Figure 22: Egger’s test of coefficient of variation (CV) between patients with type 2 diabetes mellitus (T2DM) duration ≥10 years and <10 years groups

Figure 23: Funnel plot of percentage of hexagonality (HEX) between patients with type 2 diabetes mellitus (T2DM) duration ≥10 years and <10 years groups

Figure 24: Egger’s test of percentage of hexagonality (HEX) between patients with type 2 diabetes mellitus (T2DM) duration ≥10 years and <10 years groups

Table 1 Egger's test results for publication bias across all meta-analysis outcomes.

| Parameters | Number of studies | P-value |
| --- | --- | --- |
| **T2DM vs. Controls** |  |  |
| ECD | 20 | 0.184 |
| CV | 18 | 0.056 |
| HEX | 18 | 0.057 |
| **DR vs. no DR** |  |  |
| ECD | 10 | 0.590 |
| CV | 10 | 0.764 |
| HEX | 10 | 0.721 |
| **PDR vs. NPDR** |  |  |
| ECD | 10 | 0.592 |
| CV | 10 | 0.517 |
| HEX | 10 | 0.909 |
| **Duration ≥10 years vs. <10 years** |  |  |
| ECD | 10 | 0.843 |
| CV | 8 | 0.010^*^ |
| HEX | 8 | 0.880 |

Note: ^*^ indicates P < 0.05, ^**^ indicates P < 0.01, ^***^ indicates P < 0.001;

c
