# Supplementary material for: Corneal endothelial changes in type 2 diabetes mellitus: a systematic review and meta-analysis with subgroup comparisons by diabetic retinopathy status and disease duration
Source: Front Endocrinol (Lausanne). 2026 Sep 29;17:1982467. doi: 10.3389/fendo.2026.1982467 (PMC13657926; doi:10.3389/fendo.2026.1982467)
Supplement: Supplementary file 5 [file SupplementaryFile5.docx]

**Supplementary File 5**

Figure 1: Trim-and-fill analysis of coefficient of variation (CV) between type 2 diabetes mellitus (T2DM) and healthy controls

Figure 2: Trim-and-fill analysis of percentage of hexagonality (HEX) between type 2 diabetes mellitus (T2DM) and healthy controls

 Figure 3: Trim-and-fill analysis of endothelial cell density (ECD) between proliferative diabetic retinopathy (PDR) and non-proliferative diabetic retinopathy (NPDR) groups

Figure 4: Trim-and-fill analysis of endothelial cell density (ECD) between with type 2 diabetes mellites duration ≥10 years and <10 years groups

Figure 5: Trim-and-fill analysis of coefficient of variation (CV) between patients with type 2 diabetes mellitus (T2DM) duration ≥10 years and <10 years groups

Table 1 Trim-and-fill analysis results across five meta-analysis outcomes.

| Parameters | Observed studies | Cohen's d (before) | 95%CI (before) | Observed and Imputed studies | Cohen's d (after) | 95%CI (after) |
| --- | --- | --- | --- | --- | --- | --- |
| **T2DM vs. Controls** |  |  |  |  |  |  |
| CV | 19 | 0.249 | [0.164, 0.334] | 19 | 0.249 | [0.164, 0.334] |
| HEX | 19 | -0.221 | [-0.415, -0.027] | 25 | -0.351 | [-0.527, -0.176] |
| **PDR vs. NPDR** |  |  |  |  |  |  |
| ECD | 10 | -0.209 | [-0.489, 0.071] | 11 | -0.237 | [-0.506, 0.031] |
| **Duration ≥10 years vs. <10 years** |  |  |  |  |  |  |
| ECD | 10 | -0.389 | [-0.754, -0.025] | 10 | -0.389 | [-0.754, -0.025] |
| CV | 8 | 0.148 | [-0.030, 0.327] | 12 | 0.312 | [0.126, 0.497] |
