# Supplementary material for: Corneal endothelial changes in type 2 diabetes mellitus: a systematic review and meta-analysis with subgroup comparisons by diabetic retinopathy status and disease duration
Source: Front Endocrinol (Lausanne). 2026 Sep 29;17:1982467. doi: 10.3389/fendo.2026.1982467 (PMC13657926; doi:10.3389/fendo.2026.1982467)
Supplement: Supplementary file 6 [file SupplementaryFile6.docx]

**Supplementary File 6**Figure 1: Sensitivity analysis of endothelial cell density (ECD) between type 2 diabetes mellitus (T2DM) and healthy controls.

Figure 2: Sensitivity analysis of coefficient of variation (CV) between type 2 diabetes mellitus (T2DM) and healthy controls.

Figure 3: Sensitivity analysis of percentage of hexagonality (HEX) between type 2 diabetes mellitus (T2DM) and healthy controls.

Figure 4: Sensitivity analysis of endothelial cell density (ECD) between diabetic retinopathy (DR) and no diabetic retinopathy groups

Figure 5: Sensitivity analysis of coefficient of variation (CV) between diabetic retinopathy (DR) and no diabetic retinopathy groups

Figure 6: Sensitivity analysis of percentage of hexagonality (HEX) between diabetic retinopathy (DR) and no diabetic retinopathy groups

Figure 7: Sensitivity analysis of endothelial cell density (ECD) between proliferative diabetic retinopathy (PDR) and non-proliferative diabetic retinopathy (NPDR) groups

Figure 8: Sensitivity analysis of coefficient of variation (CV) between proliferative diabetic retinopathy (PDR) and non-proliferative diabetic retinopathy (NPDR) groups

Figure 9: Sensitivity analysis of percentage of hexagonality (HEX) between proliferative diabetic retinopathy (PDR) and non-proliferative diabetic retinopathy (NPDR) groups

Figure 10: Sensitivity analysis of endothelial cell density (ECD) between patients with type 2 diabetes mellitus (T2DM) duration ≥10 years and <10 years groups

Figure 11: Sensitivity analysis of coefficient of variation (CV) between patients with type 2 diabetes mellitus (T2DM) duration ≥10 years and <10 years groups

Figure 12: Sensitivity analysis of percentage of hexagonality (HEX) between patients with type 2 diabetes mellitus (T2DM) duration ≥10 years and <10 years groups
